# Development of Advanced Macrosphelides: Potent Anticancer Agents

**DOI:** 10.3390/molecules20034430

**Published:** 2015-03-10

**Authors:** Seung-Mann Paek

**Affiliations:** College of Pharmacy and Research Institute of Pharmaceutical Sciences, Gyeongsang National University, Jinju daero, Jinju, Gyeongnam 660-701, Korea; E-Mail: million@gnu.ac.kr; Tel.: +82-55-772-2424; Fax: +82-55-772-2429

**Keywords:** macrosphelides, medicinal chemistry, analogs

## Abstract

Synthetic approaches to macrosphelide derivatives, based on medicinal chemistry, are summarized. This review contains conventional medicinal chemistry approaches, combinatorial chemistry, fluorous tagging techniques and affinity chromatography preparation. In addition, advances in their apoptosis-inducing activities are also included.

## 1. Introduction

As the urgent demand for the development of new drugs increases, rapid innovations in medicinal chemistry, natural product isolation, chemical biology approaches and target protein identification techniques have taken place [1,2,3]. In particular, new therapeutic agents for cancer patients have been anticipated because most cancer cells are resistant to long-term therapy and are readily mobile through metastasis [4]. For this reason, tremendous efforts have been poured into discovering novel metastasis inhibitors and tumor cell growth inhibitors [5]. Recent research is also attempting to discover new chimeric agents that possess tumor metastasis, growth inhibitory activities and are non-toxic to normal cells [6,7]. The macrosphelides (MS) satisfy these requirements very well.

**Figure 1:**
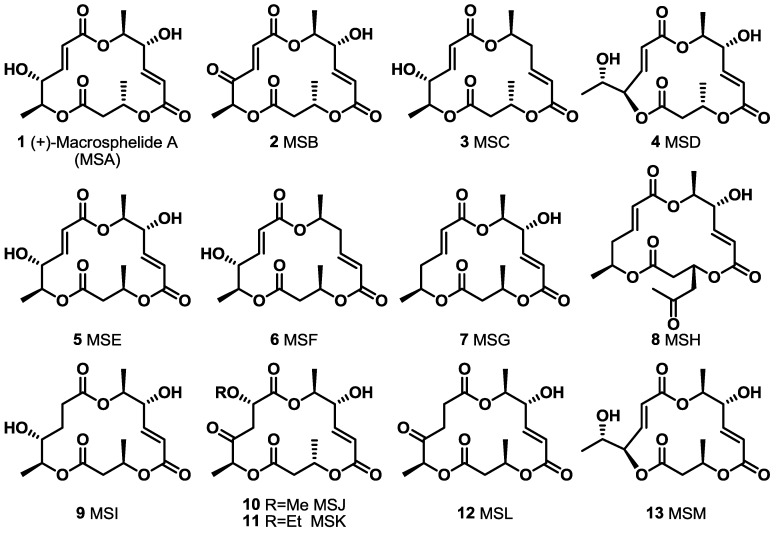
Natural Macrosphelides.

Since the first isolation of MS in 1995, this 16-membered macrolide has been of high interest to synthetic and medicinal chemists because of its non-toxicity and various biological activities ([8,9,10], Figure 1). MSA inhibits the adhesion of human leukemia HL-60 cells to human-umbilical-vein endothelial cells, shows no acute toxicity by *i.p.* injection into BDF1 mice at 200 mg/kg for 5 days, displays anticancer activity against lung metastasis of B16/BL6 melanoma in mice and even exhibits antimicrobial activity [8,11,12,13,14]. More importantly, MSB shows a strong *in vivo* immunosuppressant activity equipotent to that of rapamycin or cyclosporine, which are used clinically [15]. Based on these basic bioactivities, not only natural MS isomers [16], but also variety of MS derivatives have been synthesized to improve their activity and reveal undiscovered features [17]. In this review, a synthetic study to develop MS-related derivatives is discussed.

## 2. Results and Discussion

### 2.1. The First Unnatural Isomers of MS

**Scheme 1:**
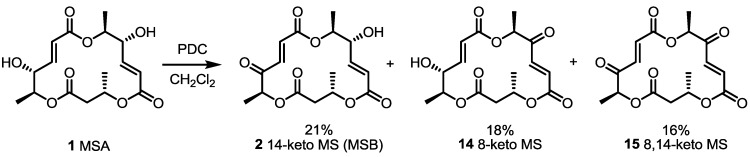
Oxidation of MSA to Other MS Analogs.

The first derivatization of MS was carried out to determine the exact structure of MSB via oxidation of MSA, as shown in Scheme 1 [11,18]. After first isolation of MSA and B from *Microsphaerosis* FO5050, MSA was treated with pyridinium dichlorochromate (PDC) to oxidize the secondary hydroxyl group to determine the absolute structure of MSB. As expected, oxidation showed non-chemoselectivity, resulting in a low yield of MSB, along with 8-keto MS **14** and diketo-MS **15** in almost equal amounts. Although these two unnatural isomers weren’t synthesized for medicinal chemistry purposes, they were utilized for structure-activity relationship (SAR) studies and further derivatization afterward.

### 2.2. Combinatorial Chemistry of MS

**Figure 2:**
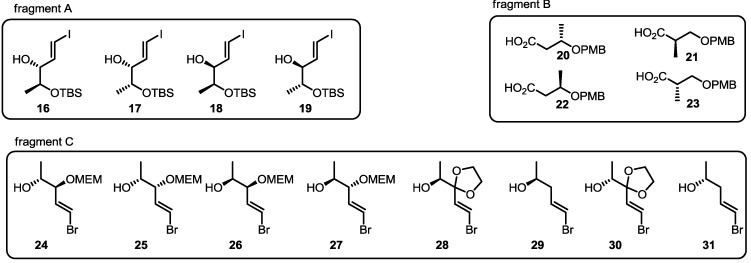
Fragments for the Combichem Approach of MS.

In the early 2000s, resin-bound preparation of an MS-focused chemical library using the carbonylative cyclization route was utilized, as shown in Figure 2 [19]. Employing conventional synthesis, three fragments were prepared for combinatorial chemistry (combichem) of MS. To maximize substructure diversity, each fragment was modified at the chiral center or the substituent of an appropriate carbon in the structure. Fragment C contained eight isotypes depending on the chiral center, oxidation state or protection group.

**Scheme 2:**
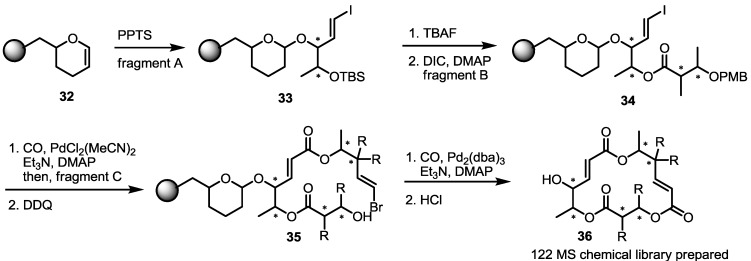
Combichem Approach for MS Derivatives.

Attachment of resin to fragment A commenced with a pyran formation reaction catalyzed by PPTS to form resin-bound fragment A **33**. (Scheme 2) Then, the vinyl iodide **33** was treated with TBAF to liberate the free hydroxyl group, which was converted into ester **34** through a coupling reaction with fragment B. Pd(0)-mediated insertion of CO into the vinyl iodide moiety and the subsequent addition of fragment C produced diester **35** after PMB deprotection with DDQ [20]. The second Pd(0)-mediated carbonylative cyclization was then executed to produce MS isomers after acidic detachment of the resin. It was noteworthy that Pd(0)-mediated CO insertion created a new C-C bond in the ester, although the catalyst was slightly different. Another feature of this reaction is that the second CO insertion was used for macrocyclization of the 16-membered macrolide skeleton in excellent yield. Before this result, Yamaguchi macrolactonization was adopted for most cases of MS synthesis [21]. More important is that this cyclization was applied to a resin-bound substrate to prepare the MS-related chemical library. Employing this synthetic strategy, 122 MS isomers, including natural and unnatural ones, were prepared simultaneously.

### 2.3. Synthesis of Core Structure and Its Derivatization

**Scheme 3:**
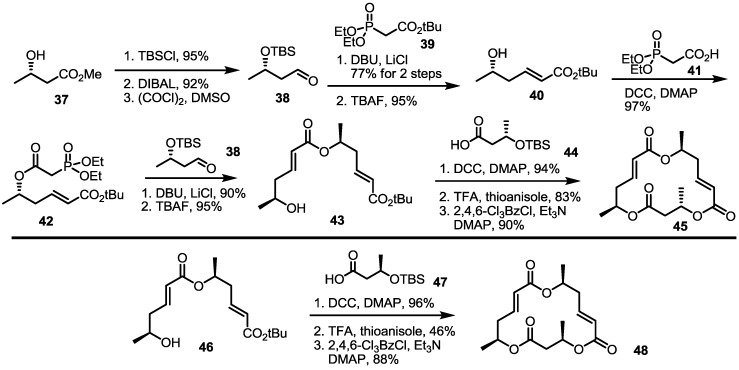
Synthesis of the MS-Core Structure.

An allylic oxidation strategy for a versatile synthesis of MS was also attempted [22]. The Nemoto group prepared a simple MS core skeleton **45** using a Horner-Emmons reaction/esterification route, as shown in Scheme 3. Hydroxybutyrate **37** was converted into aldehyde **38** via TBS protection and a semi-reduction sequence. The aldehyde **38** was then transformed into **40** through a Horner-Emmons reaction followed by deprotection. For the second homologation, a phosphonate **42** was prepared by esterification with phosphocarboxylic acid **41** to produce hydroxyester **43** after a Horner-Emmons reaction with aldehyde **38** and TBS deprotection. Finally, the core skeleton **45** was synthesized in excellent yield by esterification with **44**/acidic treatment/Yamaguchi lactonization. Using the same protocol, the diastereomeric MS core **48** was also prepared as shown below.

**Figure 3:**
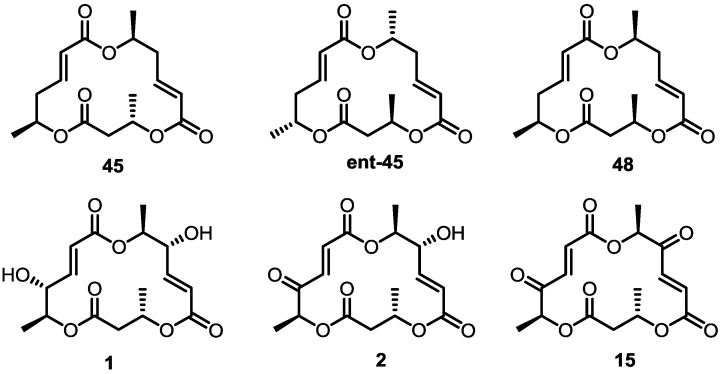
MS and Modified MS Core Skeleton.

These MS core isomers directly provided basic information on the structure-activity relationship (SAR) when compared to previously synthesized isomers [22]. MS core products all showed a slight inhibition effect on cell proliferation at low concentrations, while having a significant enhancement on the same cells at high concentrations. A cytotoxic effect of carbonylated MSB **2** and diketo-MS **15** was also observed at high concentrations, while MSA **1** didn’t display any cytotoxicity. It is thought that the carbonyl group, rather than the hydroxyl group at C14 or C8, may be connected to the cytotoxicity of MS (Figure 3).

More impressive results came with the apoptosis-inducing activity of the MS isomers. After collaboration with the Kondo Group [23], MSB **2** and diketo-MS **15** were found to induce mild apoptosis in human lymphoma U937 cells via the Fas/caspase-8-dependent pathway. This comparison might indicate that the 14-carbonyl group is crucial for programmed cell death. It was also reported that this apoptosis induction could be enhanced under mild hyperthermia conditions [24].

**Scheme 4:**
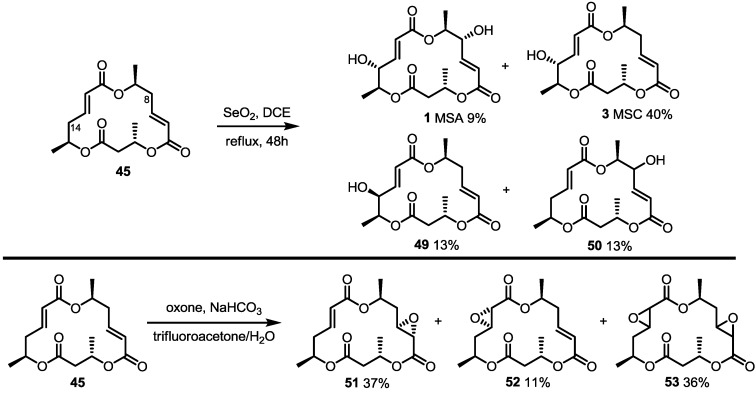
Direct Oxidation of the MS Core Skeleton.

With the core structure of MS, allylic oxidation leading to the derivatization of MS was also attempted [25]. Because the X-ray structure of the MS core showed a complete shield from the β-face of C8, while a relatively open α-face at C14, it was anticipated that the C14 α-hydroxyl group would be introduced as a major product. (Scheme 4) Actually, when **45** was treated with SeO_2_ in refluxing dichloroethane, the expected oxidation product **3** (MSC) was obtained in 40% yield, together with di-oxidized product **1** (MSA), the C14 epimeric product **49** and an unidentified product **50**. Similarly, epoxidation of a conjugated alkene also produced a β-epoxide as the major product, although the C6-C7 alkene was more reactive than the C12-C13 alkene. Employing this direct oxidation strategy, a variety of MS isomers could be easily prepared.

### 2.4. Modification of the MS Structure for Detailed SAR Studies

**Scheme 5:**
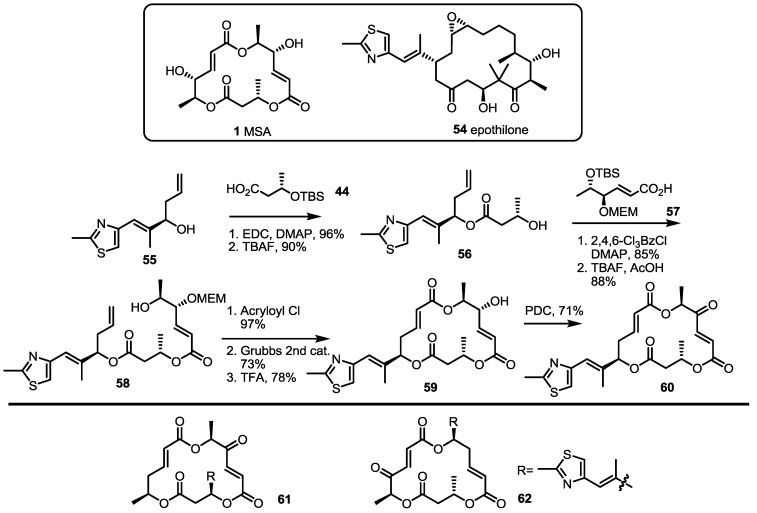
Preparation of MS-Epothilone Hybrid Agents.

More dramatic advances were accomplished through preparation of chimeric molecules with an MS skeleton and epothilone side chain (Scheme 5) [26]. Inspired by epothilone’s extraordinary cytotoxicity to broad cancer cell lines and its 16-membered macrolide structure, just like MS, molecular design of the MS-containing thiazole side chain of epothilone was pursued to develop more potent MS derivatives than natural ones [27]. To validate this hypothesis, the known homoallylic alcohol **55** was esterified and deprotected to hydroxyester **56**, which was converted into diester **58** through iterative esterification/TBS deprotection [28]. As illustrated in the previous synthesis of MSA, B and E, the macrolide framework was constructed using the ring-closing metathesis (RCM) route after acryloylation/RCM/MEM deprotection in good yield [29]. Finally, oxidation of the secondary hydroxyl group produced the desired ketone **60** [30]. Using the same protocol, a thiazole side chain could be successfully introduced at the C3 or C9 position.

The apoptosis-inducing activity of the chimeric agents was tested. [26] While diketo-MS **15** shows slight apoptosis-inducing activity, the hybrid molecule **60** exhibited significant apoptosis-inducing activity against the U937 cell line at a concentration of 1 μM. It is also impressive that hybrid **60** didn’t show necrosis at the same concentration. This dramatic and successful improvement of the intrinsic activity in MS has influenced further research into the medicinal chemistry and the development of related molecules.

Fascinated by these outstanding advances, mechanistic investigation of **60**-induced apoptosis was also carried out through a collaboration of the Kondo and Nemoto Groups [31]. They demonstrated that **60** caused rapid ROS generation and mitochondrial dysfunction in U937 cells, triggering the mitochondria-dependent apoptosis pathway. They also showed that **60** is potent against human colon carcinoma (HCT116) and gastric cancer (ASG) cells, while leaving normal fibroblasts intact.

**Scheme 6:**
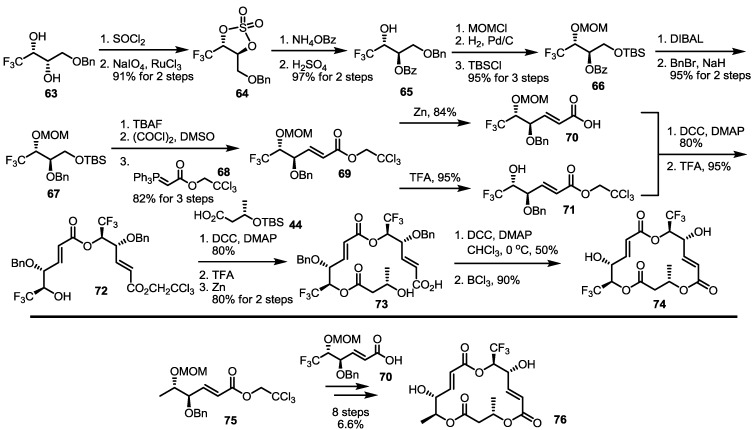
Preparation of CF_3_-substituted MS **74** and **76**.

Trifluoromethylated derivatives were also prepared [32,33]. To diversify the electronic environment of the MS skeleton, a CF_3_ group was designed to replace the terminal CH_3_ group, as shown in Scheme 6 [34]. A CF_3_-substituted diol **63** was converted into benzoylated diol **65** via inversion of the chiral center [35]. After protection group exchange, TBS ether **67** was treated in three steps of TBS deprotection/Swern oxidation/Wittig olefination to produce the conjugated ester **69** in 82% yield. This common intermediate **69** was then transformed into carboxylic acid **70** and free alcohol **71** to provide dimeric ester **72** via esterification and MOM deprotection. Finally, an iterative esterification/deprotection/lactonization sequence resulted in CF_3_-substituted MS **74** in good yield. Instead of CF_3_-substituted alcohol **71**, CH_3_-substituted alcohol **75** was also used to prepare mono-CF_3_-substituted MS **76**, following the established 8-step route.

**Scheme 7:**
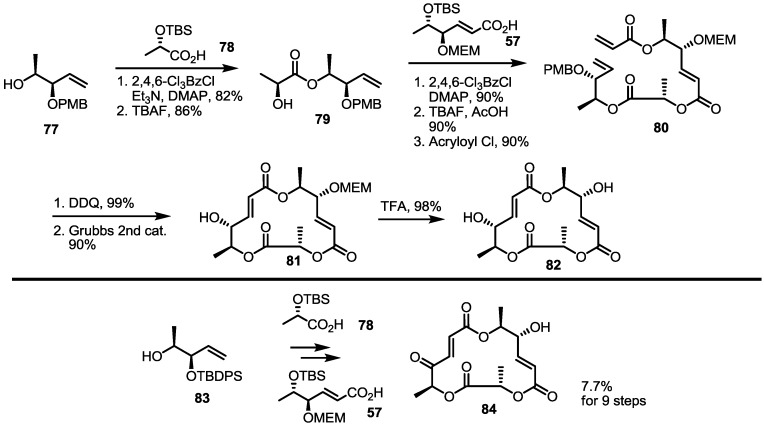
MS Derivatives Possessing a 15-membered Macrolide Skeleton.

The ring size of MS was also changed to investigate the SAR thoroughly, as shown in Scheme 7 [36]. Known homoallylic alcohol **77** and carboxylic acid **78** were coupled to produce ester **79** after TBS deprotection [29]. A second esterification of **79** with carboxylic acid **57**, followed by deprotection and acryloylation, produced acrylate **80**. As previously reported, PMB deprotection and the RCM sequence produced MEM-protected MS derivative **81**, which could be changed to the desired 15-membered lactone **82** in yield of 98%. The 15-membered MSB analog **84** was also prepared using the same synthetic procedure and TBDPS-protected starting material for practical reasons in large-scale synthesis.

**Scheme 8:**
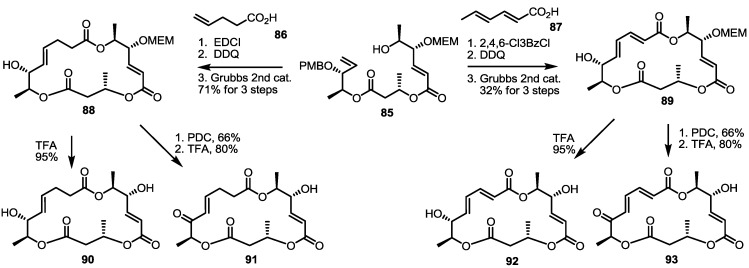
Ring-enlarged MS Derivatives.

Together with the ring-contracted derivatives, ring-enlarged molecules were also pursued [37]. (Scheme 8) The known synthetic intermediate **85** was esterified using carboxylic acid **86** or **87** and converted into the 18-membered macrolide **88** or **89** via PMB deprotection and RCM [29]. Acidic deprotection or oxidation followed by deprotection produced **90**–**93** in moderate yield.

**Scheme 9:**
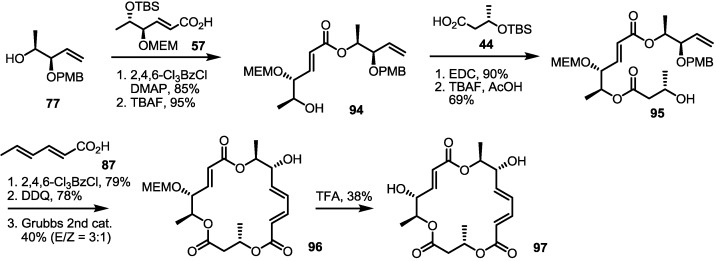
Ring-enlarged MS Derivatives with the Lower Part Changed.

The ring size was also modified at the O16-O4 moiety of the MS skeleton (Scheme 9). PMB-substituted alcohol **77** was converted into an advanced intermediate **95** via iterative esterification/deprotection sequence [29]. Then, the secondary hydroxyl group of **95** was esterified using sorbic acid to produce macrolactone **96** after the established deprotection/RCM route, although it produced an *E*/*Z* mixture in low yield. They were separated after final MEM deprotection.

**Scheme 10:**
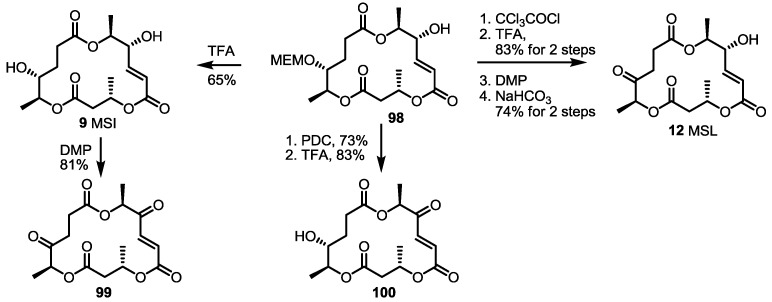
Synthesis of Dihydro Derivatives of MS.

The oxidation state of MS was also modified, as shown in Scheme 10 [37]. The known intermediate **98** was converted into versatile derivatives **99**, **100** via selective functionalization. Simple acidic deprotection gave MSI itself, which could be oxidized to the diketone molecule **99**. Compound **98** produced **100** or **12** by oxidation/deprotection or the protection group exchange/oxidation/deprotection sequence.

**Scheme 11:**
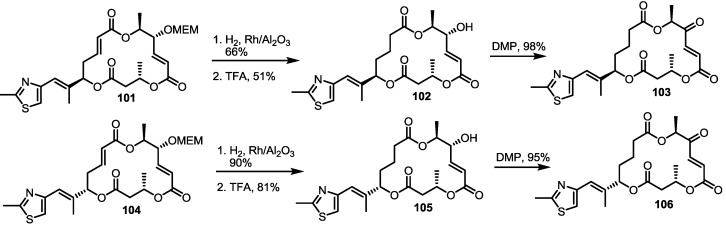
Direct Saturation of Olefin in Thiazole-substituted MS.

To elucidate the role of electron-deficient alkenes, hydrogenated MS derivatives were also prepared from the thiazole-substituted intermediate **101** (Scheme 11). It was found that reduction and acidic deprotection produced the reduced-thiazole derivatives **102** [27]. Final oxidation also resulted in another important MS derivative. Application of the C15-epmieric intermediate **104** also produced the C15-epimeric final product **106**.

**Scheme 12:**
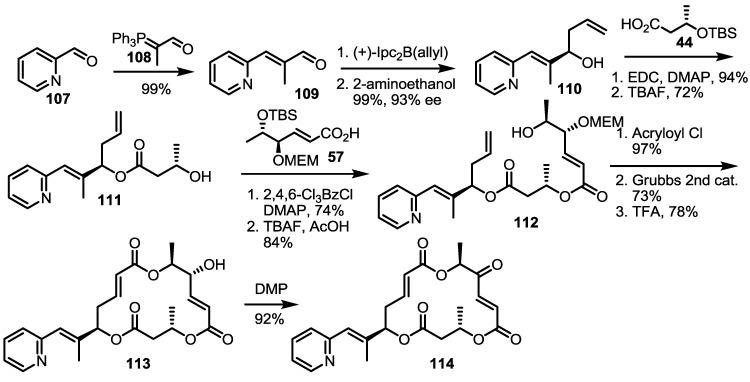
Pyridine-substituted MS Derivatives.

In the same paper, pyridine-substituted MS was also prepared instead of a thiazole side chain [37] (Scheme 12). Application of the Wittig reaction and Brown allylation to a formylpyridine **109** produced homoallylic alcohol **110**, which was converted into hydroxyester **111** via esterification and deprotection. After iterative esterification/deprotection, the resulting hydroxyester **112** was cylized to macrolactone **113** using the acryloylation/RCM/deprotection sequence. Finally, oxidation of the free hydroxyl group led to the desired ketone in excellent yield.

**Scheme 13:**
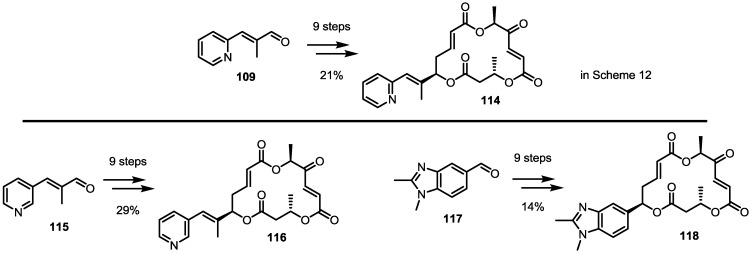
Other Heterocycle-substituted MS Derivatives.

Impressed with the potency of the heterocycle-combined MS, other heterocycles were also attached to the MS skeleton simultaneously (Scheme 13) [38]. In 2010, the Nemoto Group reported that 3-pyridyl-MS **116** was almost equipotent to 2-pyridyl-MS **114** in terms of apoptosis of U937 cells [38]. However, benzimidazole-substituted MS **118** didn’t exhibit apoptotic activity. These conflicting results indicate that there are still a lot of work to be done to optimize the SAR of MS.

**Scheme 14:**
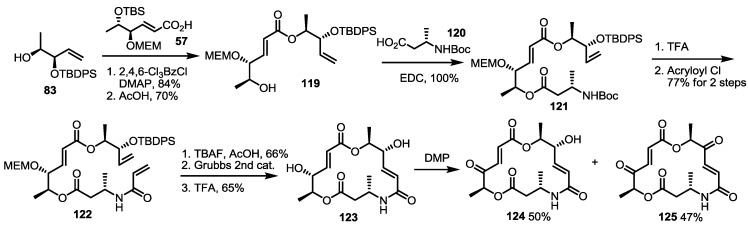
Preparation of Aza-MS **124** and **125**.

The skeleton itself was also changed by substitution of an ester linkage to the amide moiety, as shown in Scheme 14 [39]. Homoallylic alcohol **83** was converted into Boc-amino ester **121** via iterative esterification and the deprotection sequence in good yield [40]. Then, the Boc group was deprotected to give the corresponding amide after acryloylation of the amino group. TBDPS deprotection, followed by RCM and MEM deprotection, produced amino-MS. Keto-amino-MS derivatives were also prepared using non-selective oxidation with Dess-Martin Periodinane treatment.

**Scheme 15:**
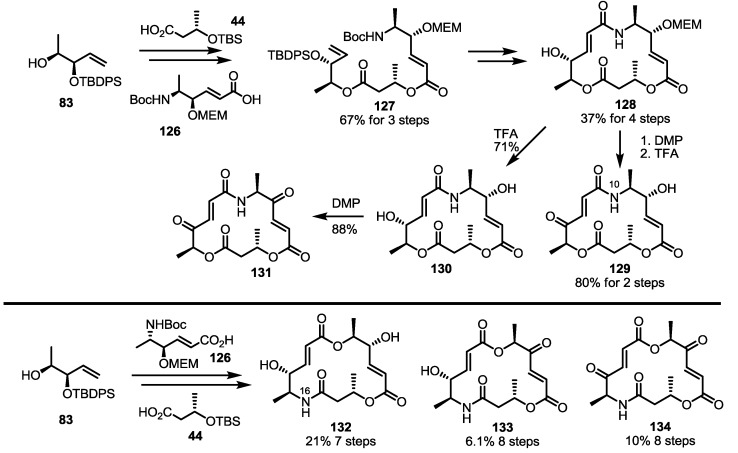
Preparation of Other Aza-MS **129**–**134**.

Using the established procedure to introduce nitrogen into the MS skeleton, the other ester moiety was also replaced by an amido group, as shown in Scheme 15 [39]. As for the preparation of MS **123** above, homoallylic alcohol **83** could be converted into Boc-amino ester **127** through sequential esterification with **44** and **126**. This building block could also be converted into N10-MS in moderate yield. Finally, deprotection or the oxidation strategy produced the target N-10 MS derivatives **129**–**131** very well. N-16 MS **132**–**134** were also prepared using well-designed esterification of the three building blocks and RCM.

**Scheme 16:**
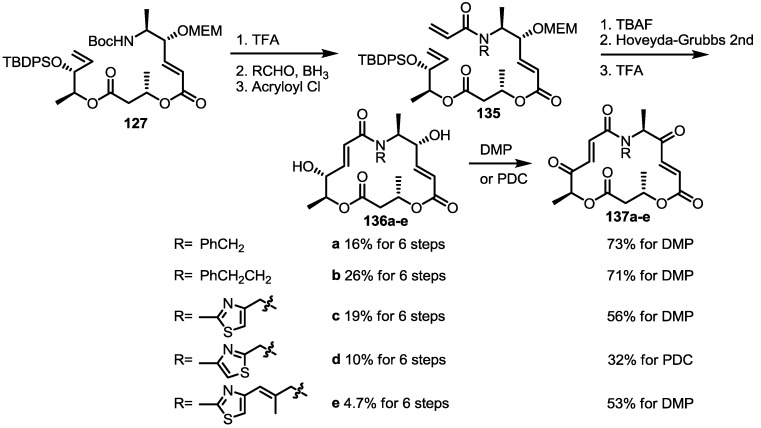
Synthesis of N-alkylated Derivatives.

Replacement of oxygen with nitrogen results in additional hydrogen bonding (NH) forming spontaneously [39]. For exact SAR, it still requires exclusion of hydrogen bonding because this molecular interaction may distort its own 3D structure. N-alkylated MS derivatives must be prepared for this reason and their preparations are summarized in Scheme 16.

The previous synthetic intermediate **127** was treated with TFA to obtain the free amino group that would be used for reductive amination and acryloylation to produce acrylamide **135**. Well-established silyl deprotection/RCM/MEM deprotection produced N-alkyl MS **136** of various sizes and heterocyclic substituents. Additional oxidation also resulted in di-keto amino derivatives **137** in moderate yield.

**Scheme 17:**
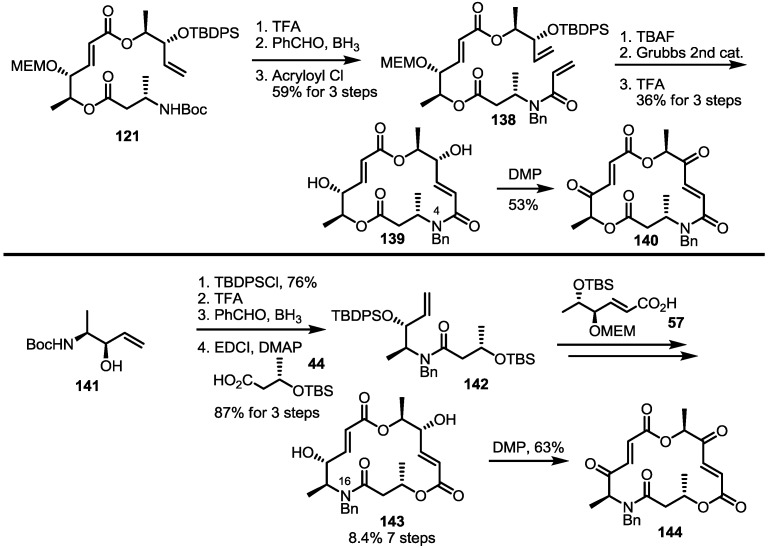
Synthesis of Other N-alkylated Derivatives.

Another previous intermediate **121** could also be converted into other valuable aminoalkyl products **139** and **140** via the intermediate **138** (Scheme 17) [39]. The starting material **121** was transformed into the N4-aminoalkyl product **139** in 6 steps via the previously established synthetic procedure (RCM route). Dess-Martin oxidation also produced the corresponding keto product **140** in moderate yield. Employing a similar synthetic route and starting material **141**, N16-aminoalkyl MS **143**, **144** were also obtained in moderate yield as shown below.

**Scheme 18:**
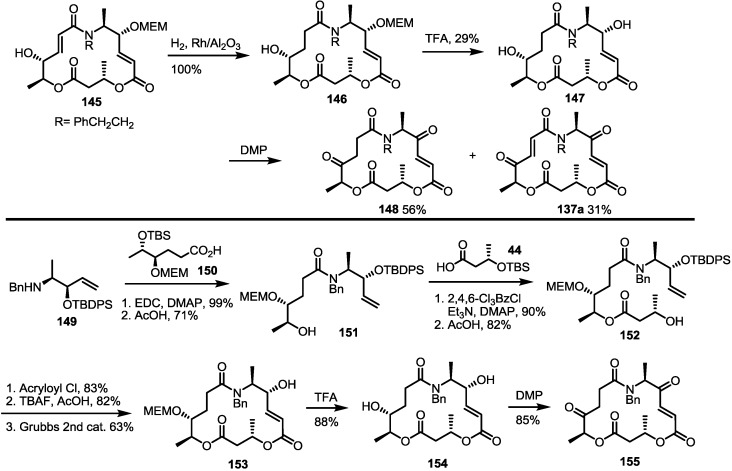
Synthesis of Dihydro-aza MS Derivatives.

The amino derivatives were also used to prepare dihydro-aza MS **148**, as shown below (Scheme 18) [41]. The previous synthetic intermediate **145** underwent hydrogenation to produce a chemoselective reduction product **146**, which was converted into **147** via MEM deprotection. Interesting is that the unsaturated derivative **137a** was also regenerated during preparation of the keto MS **148** via DMP oxidation. N10-amino dihydro MS couldn’t be synthesized from the corresponding alkene **128**. Therefore, modification of the early steps was necessary. Actually, the benzylamino alkene **149** was coupled with hexanoic acid **150** to give amido alkene **151** after acidic TBS deprotection [37]. Routine esterification/deprotection also produced hydroxyl ester **152**, which could be changed to macrolactam **153** via the acryloylation/deprotection/RCM route. Finally, TFA-mediated deprotection and oxidation gave the desired products **154** and **155**, respectively.

### 2.5. Fluorous Tagging Strategy for Diverse Synthesis of MS Stereoisomers

**Scheme 19:**
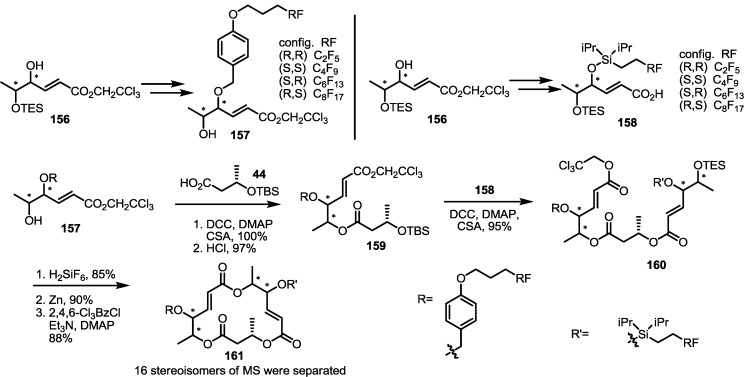
Fluoride Tagging Method for MS Derivatives Synthesis.

In 2012, the Curran group reported that the application of a fluorous tagging technique to MS research may allow complete synthesis of all MS stereoisomers through one synthetic route (Scheme 19) [42]. A common intermediate **156**, prepared from a traditional synthetic method, was protected with a PMB group that contains a fluoride tail of varied size. Similarly, TIPS-protected carboxylic acid **158** were also prepared using different fluorous alkyl tails. Then, esterification of the two building blocks was performed using carboxylic acid **158** and alcohol **159**, prepared from PMB ether **157** via esterification with simple carboxylic acid **44** and acidic deprotection, to produce trimeric ester **160** in 95% yield. Finally, TES deprotection, ester hydrolysis and Yamaguchi lactonization produced a mixture of all 16 stereoisomers together. Because these isomers possess different numbers of fluoride tails, they could be separated efficiently. Employing this strategy, Curran group tried to confirm the exact structure of MSD and discovered that MSD is not a stereoisomer, but rather a regioisomer of MS.

**Scheme 20:**
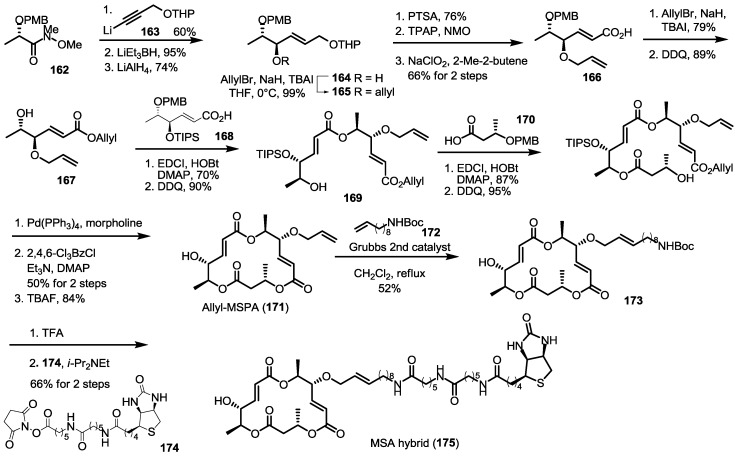
Preparation of MS-Affinity Chromatography.

### 2.6. Preparation of MS-Biotin Hybrid

Recently, MS-based affinity chromatography was performed as shown in Scheme 20. Hirao alkynylation of the readily available Weinreb amide **162** [43,44], followed by double reduction afforded the chiral secondary alcohol **164** efficiently. After conventional protection/deprotection and oxidation, allyl ester **167** and carboxylic acid **168** could be esterified to **169** after PMB deprotection. Iterative esterification/deprotection sequence produced allyl-MSA **171** in good yield. With this pivotal synthetic platform in hand, aminoalkyl side chain was homologated into carbamate **173** employing the cross metathesis. Finally, acidic Boc deprotection and amidation with commercially available biotin analog **174** afforded MS-biotin hybrid **175**. This synthetic hybrid is expected to show biological process in biochemically more detailed view.

### 2.7. Biological Assay

Among all derivatives of MS, some of them have been tested for their biological profile and SAR study. Some of their impressive data are listed below.

Table 1 shows the prepared hybrid of MS and epothilone, a promising apoptosis-inducing agent, could improve the intrinsic activity of MS itself [26]. It is interesting to note that the same introduction at the C9 position didn’t produce a compound as potent as the C15 hybrid **60**. After this result, extensive modifications of the side chain of MS were carried out.

**Table 1:** Apoptosis Inducing Activity of MS Derivatives ^a,b,c^.

Derivative	Activity	Conc.	Derivative	Activity	Conc.
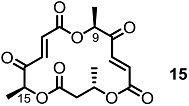	<1%	10 μM	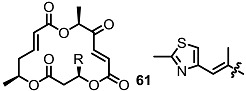	4%–5%	1 μM
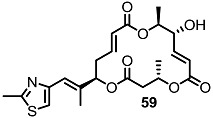	<1%	1 μM	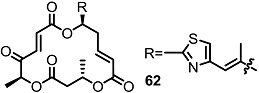	<1%	1 μM
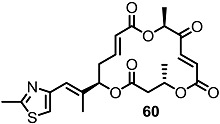	>10%	1 μM			

^a^ Human lymphoma U937 cells were treated at various concentration (1–10 μM); ^b^ Apoptosis assessment was carried out by flow cytometry of annexin V/FITC and propidium iodide staining cells; ^c^ Activity was determined with fraction of cells.

Impressed with the promising apoptosis-inducing activity of MS derivatives, a more detailed SAR study was executed as shown in Table 2 [37]. Because the C15 hybrid showed more potent activity than **60** in Table 1, dihydro- or ring-enlarged MS derivatives were developed. However, this modification didn’t afford more improved apoptosis-inducing activity. It seems this modification could alter the conformation of the MS skeleton.

**Table 2:** Apoptosis Inducing Activity of MS Derivatives Based on DNA Fragmentation ^a,b^.

Derivative	Activity	Conc.	Derivative	Activity	Conc.
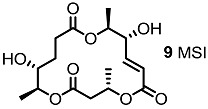	<10%	10 μM	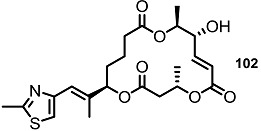	<10%	10 μM
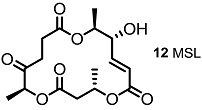	<10%	10 μM	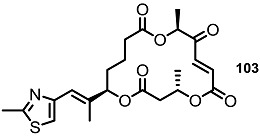	20%	10 μM
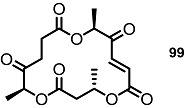	>30%	10 μM	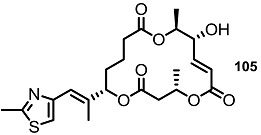	<10%	10 μM
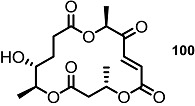	25%	10 μM	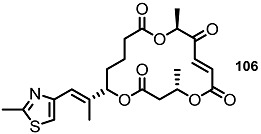	30%	10 μM
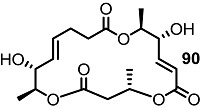	10%	10 μM	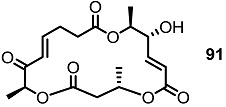	25%	10 μM
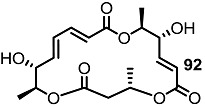	>10%	10 μM	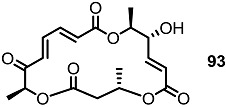	>10%	10 μM

^a^ Human lymphoma U937 cell s were treated at 1–10 μM concentration; ^b^ Activity was determined with DNA fragmentation.

Nitrogen substituted MS derivatives and their apoptosis-inducing activity are shown in Table 3 [39]. Other than the increase in activity in the diketo derivatives **137** compared to the dihydroxy derivatives **136**, precise SAR in **137** is difficult to conclude yet. This means more advances in the medicinal chemistry research on MS is still necessary for *in vivo* and clinical usage.

**Table 3:** Apoptosis Inducing Activity of aza-MS Derivatives Based on DNA Fragmentation ^a,b^.

Derivative	Activity	Conc.	Derivative	Activity	Conc.
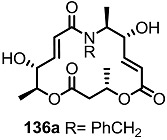	<3%	10 μM	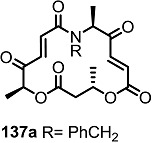	<3%	10 μM
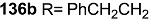	<3%	10 μM	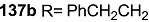	43%	10 μM
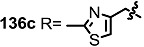	<3%	10 μM	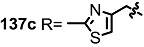	7%	10 μM
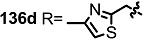	<3%	10 μM	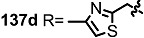	6%	10 μM
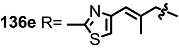	<3%	10 μM	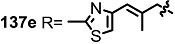	16%	10 μM
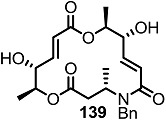	<3%	10 μM	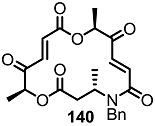	<3%	10 μM
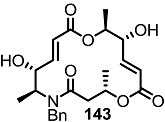	<3%	10 μM	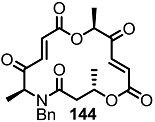	22%	10 μM

^a^ Human lymphoma U937 cell s were treated at 1–10 μM concentration; ^b^ Activity was determined with DNA fragmentation.

## 3. Conclusions

As a promising anticancer agent, MS has been focused on because of its non-toxicity, inhibitory activity for tumor metastasis, apoptosis-inducing activity and other biological activities. However, its low potency has hampered efficient development of a MS-based medicine or candidate. To overcome this hurdle, a variety of research has been carried out, as discussed in this review. Based on the results accumulated to date, hopefully, new discoveries should accelerate developments, leading to the successful production of more valuable small molecules in the near future.
